# Morpho-Molecular Approach Reveals Three Novel *Cladosporium* Species Associated with *Camellia sinensis* cv. *Fenghuang Dancong* in China

**DOI:** 10.3390/jof12080598

**Published:** 2026-08-11

**Authors:** Mengyi Sun, Yehui Tu, Senouwa Segla Koffi Dossou, Mengmeng Wang, Wei Li

**Affiliations:** 1 Guangdong Provincial Key Laboratory of Marine Biotechnology, Shantou University, Shantou 515000, China; 2 Guangdong Engineering Technology Research Center of Offshore Environmental Pollution Control, Shantou University, Shantou 515063, China; 3 Shantou Key Laboratory of Marine Microbial Resources and Interactions with Environment, Shantou University, Shantou 515000, China

**Keywords:** *Capnodiales*, fungal taxonomy, new species, plant-associated fungi, phylogeny

## Abstract

The genus *Cladosporium* represents one of the largest genera of dematiaceous hyphomycetes, which are commonly found on plants. The species comprising this group predominantly function as plant endophytes, producing important bioactive compounds, or act as pathogens damaging crops of significant economic interest. Nevertheless, their association with *Camellia* spp. remains to be fully elucidated, with only seven recorded cases mentioning three known species. During an investigation into fungi associated with tea plant, several *Cladosporium* isolates were obtained from symptomatic leaves of *Camellia sinensis* cv. *Fenghuang Dancong*. Using a polyphasic approach that combined morphological observations with multi-locus phylogenetic analyses based on the internal transcribed spacer (ITS), actin (act), and translation elongation factor 1-α (tef1-α), these isolates were identified as three new species in the *C. cladosporioides* complex, namely *C. dancongensis* sp. nov., *C. fenghuangensis* sp. nov., and *C. leptosporum* sp. nov. These new species can be distinguished from their close relatives (e.g., *C. benschiae*, *C. dracaenae*, *C. kuwanaspidi*, and *C. tenuissimum*) by distinct morphological features such as conidial dimensions, mycelial width, and colony characteristics. This study expands the understanding of fungi associated with *Camellia sinensis* and is the first case in which new *Cladosporium* species inhabit tea plants. Moreover, it will facilitate further research into the phylogenetic relationships of these new species and their role relative to tea plant disease.

## 1. Introduction

The genus *Cladosporium*, established in 1816 [1], is one of the most extensive and heterogeneous genera of dematiaceous hyphomycetes [2,3,4,5], with a catalogue of more than 950 names (MycoBank database, https://www.mycobank.org/; accessed on 19 June 2026). This genus is characterised by the presence of conidia in acropetal chain with a coronate scar structure, and the occurrence of *Davidiella* teleomorphs [6]. The taxonomy of this group has been the subject of extensive study over the past few decades employing polyphasic approaches. The findings of these studies indicate that this genus should be classified within a separate family, namely the *Cladosporiaceae*, which is sister to *Mycosphaerellaceae* and *Teratosphaeriaceae*, residing in the *Capnodiales* (*Dothideomycetes*) [7,8,9,10,11]. Members in this genus have been categorised into three major species complexes: *C. cladosporioides*, *C. herbarum* and *C. sphaerospermum* [4,6,12,13,14,15]. These complexes can be distinguished by their conidiogenous structures, which include unbranched or branched, nodose or non-nodulose conidiophores, and smooth, verruculose-rugose, minutely verruculose, verrucose, echinulate or spiny conidia [4,6,16].

*Cladosporium* species are ubiquitous and can be isolated from a variety of substrates, including air, soil, water, plant debris, and living plant tissues [4,17]. The organisms under consideration occupy diverse ecological niches, primarily acting as saprobes, endophytes and pathogens of plants, animals and hyperparasites of other fungi [18,19,20]. Research has demonstrated that they are predominantly distributed on plants, with a wide variety of species identified in the core microbiome of numerous plants [21]. It is noteworthy that the USDA Fungal Databases (https://fungi.ars.usda.gov/; accessed on 19 June 2026) have documented more than 1000 host genera, which represent over 8500 records. These include ecologically important cereal (e.g., corn, rice, and wheat) [4,22,23,24] and cash crops (e.g., citrus, soybean, and sugarcane) [3,16,21]. Some *Cladosporium* species hold considerable significance in the context of agricultural research, primarily due to their profound influence on the economic value of significant crops. These species have been identified as either pathogens or PGPEs (plant growth-promoting endophytes) [21,25]. However, the diversity and distribution of *Cladosporium* on *Camellia* spp. remain the subject of only a small number of investigations. Only *C. britannicum*, *C. cladosporioides* and *C. herbarum* have been found on *Camellia japonica* from China [26], the Czech Republic [3] and Japan [27] and *C. herbarum* on *Camellia sinensis* from Korea [28] and Japan [29].

*Camellia sinensis* cv. *Fenghuang Dancong* is a premium cultivar of Oolong tea that is primarily cultivated in the Fenghuang Mountain region of Guangdong Province in China. It has a long cultivation history and is renowned for its unique, natural, floral aroma and diverse flavours [30]. In recent years, the local tea industry has suffered from a serious outbreak of leaf spot diseases caused by fungi. Furthermore, the appearance and taste of the tea have been compromised, leading to economic losses for the industry. Nevertheless, the identification of the pathogenic fungi that cause these diseases has not yet been achieved. *Bipolaris bicolor* is the sole fungus for which symptoms including a yellow halo surrounding a central depression and dark brown round spots have been documented [31]. Consequently, it is hypothesised that tea plants are associated with a diverse array of fungi, including a number of previously undescribed species.

The accurate identification of fungi associated with tea plants and the clarification of the etiological agents of diseases is dependent on conducting systematic diversity and taxonomic studies. It is important to note that the genus *Cladosporium* is a core component of the plant microbiome. However, the species boundaries of this genus are often cryptic and require molecular data for reliable resolution [4,6]. Therefore, traditional morphological identification alone is insufficient. Over the past three years, our research group has carried out systematic sample collections from tea plant tissues across the core distribution area of *Camellia sinensis* cv. *Fenghuang Dancong*. Several fungal strains were isolated from necrotic lesions and initially identified as *Cladosporium* based on their coronate conidiogenous loci [4]. Subsequently, multi-locus phylogenetic analyses based on ITS-act-tef1-α revealed that these isolates formed distinct and well-supported lineages within *Cladosporium*. These clades were clearly separated from all known species, suggesting they might represent previously undescribed taxa. The objectives of the present study were to (1) accurately identify and describe these tea-associated *Cladosporium* species and (2) provide detailed illustrations of the new species.

## 2. Materials and Methods

### 2.1. Sample Collection and Fungal Isolation

Leaves of the *Camellia sinensis* cv. *Fenghuang Dancong* variety that exhibited symptoms of leaf spot were collected from tea plantations in Fenghuang Mountain, Chaozhou City, Guangdong Province, China. During the process of sampling, leaves from the same tree exhibiting comparable symptoms were meticulously placed in a sterile sampling bag, subsequently stored within a 4 °C vehicle refrigerator, and expeditiously returned to the laboratory as soon as possible. The process of fungal isolation was accomplished within the stipulated timeframe of 24 h. Two isolation methods were employed, depending on the presence of fungal structures. For the isolation of visible conidiomata, mycelia, or synnemata, the direct isolation method was employed, whereby the fungal structures were meticulously picked under an Olympus SZX7 stereomicroscope and subsequently inoculated onto potato dextrose agar (PDA) plates. For regions devoid of discernible structures, the tissue isolation method was applied [32]. Briefly, small segments (5 mm^2^) were cut from the margin of the leaf spots as well as from healthy sections. Thereafter, they were subsequently immersed into 75% ethanol for 1 min, 3.25% sodium hypochlorite for 3 min, and 75% ethanol for 30 s. Finally, they were rinsed three times in sterile water. The sterilised segments were placed onto 1/10 PDA plates containing ampicillin and streptomycin (50 mg/L each) [33]. At the same time, 100 μL of the final rinse water was also spread onto 1/10 PDA plates as a control to confirm the effectiveness of the surface sterilisation process.

All plates were incubated around 25 °C and checked daily. Individual colonies were picked up with a sterilised needle and transferred onto fresh PDA plates [34]. Representative strains are currently preserved in sterile water at 4 °C in Wei Li’s personal culture collection (WL). Type specimens of new species were deposited in the Fungarium of the Institute of Microbiology (HMAS), with the ex-type living cultures in the China General Microbiological Culture Collection Center (CGMCC).

### 2.2. Morphological Characterisation

The characteristics of each colony were examined on four different media, including PDA, malt extract agar (MEA), and synthetic nutrient-poor agar (SNA) [35]. To ensure standardised growth, isolates were first activated on PDA, then transferred to water agar (WA), and finally inoculated onto the three types of test media.

The culture characteristics, including the morphology, pigmentation, and odour of the colonies, were observed after seven days of incubation at room temperature. Colours were assessed according to the colour charts of Kornerup and Wanscher [36]. Micromorphological characteristics were examined and photo-documented using water as a mounting medium under an Olympus BX53 microscope with differential interference contrast (DIC) optics. For each species, 30 conidiophores, 30 conidiogenous cells, and 50 conidia were mounted and measured randomly, respectively.

### 2.3. DNA Extraction, PCR Amplification and Sequencing

Genomic DNA was extracted from fresh mycelia using the CTAB method [37]. Three loci, including the internal transcribed spacer (ITS), actin (act), and translation elongation factor 1-α (tef1-α) were amplified by polymerase chain reaction (PCR). The primers used were ITS5 (5′-GGAAGTAAAAGTCGTAACAAGG-3′)/ITS4 (5′-TCCTCCGCTTATTGATATGC-3′) (ITS), ACT-512F (5′-ATGTGCAAGGCCGGTTTCGC-3′)/ACT-783R (5′-TACGAGTCCTTCTGGCCCAT-3′) (act), and EF1-728F (5′-CATCGAGAAGTTCGAGAAGG-3′)/EF1-986R (5′-TACTTGAAGGAACCCTTACC-3′) (tef1-α) [38,39].

The total volume for PCR amplification was 30 μL, containing 15 μL of 2 × Rapid Taq Plus Master Mix (Dye Plus) (Vazyme; Nanjing, China), 1 µL of each primer (0.1 µM), 1 µL of genomic DNA (1–10 ng), and 12 µL of ddH2O. The PCR amplifications of ITS, act and tef1-α were set as follows: an initial denaturation at 95 °C for 5 min, followed by 35 cycles of denaturation at 95 °C for 1 min, annealing at 52 °C (ITS), 58.5 °C (act), and 55 °C (tef1-α), respectively, and extension at 72 °C for 1 min, with a final extension step at 72 °C for 10 min. PCR products obtained from three loci were purified and shipped to SinoGenoMax (Beijing, China) for Sanger sequencing in both directions.

### 2.4. Phylogenetic Analyses

**Figure 1:**
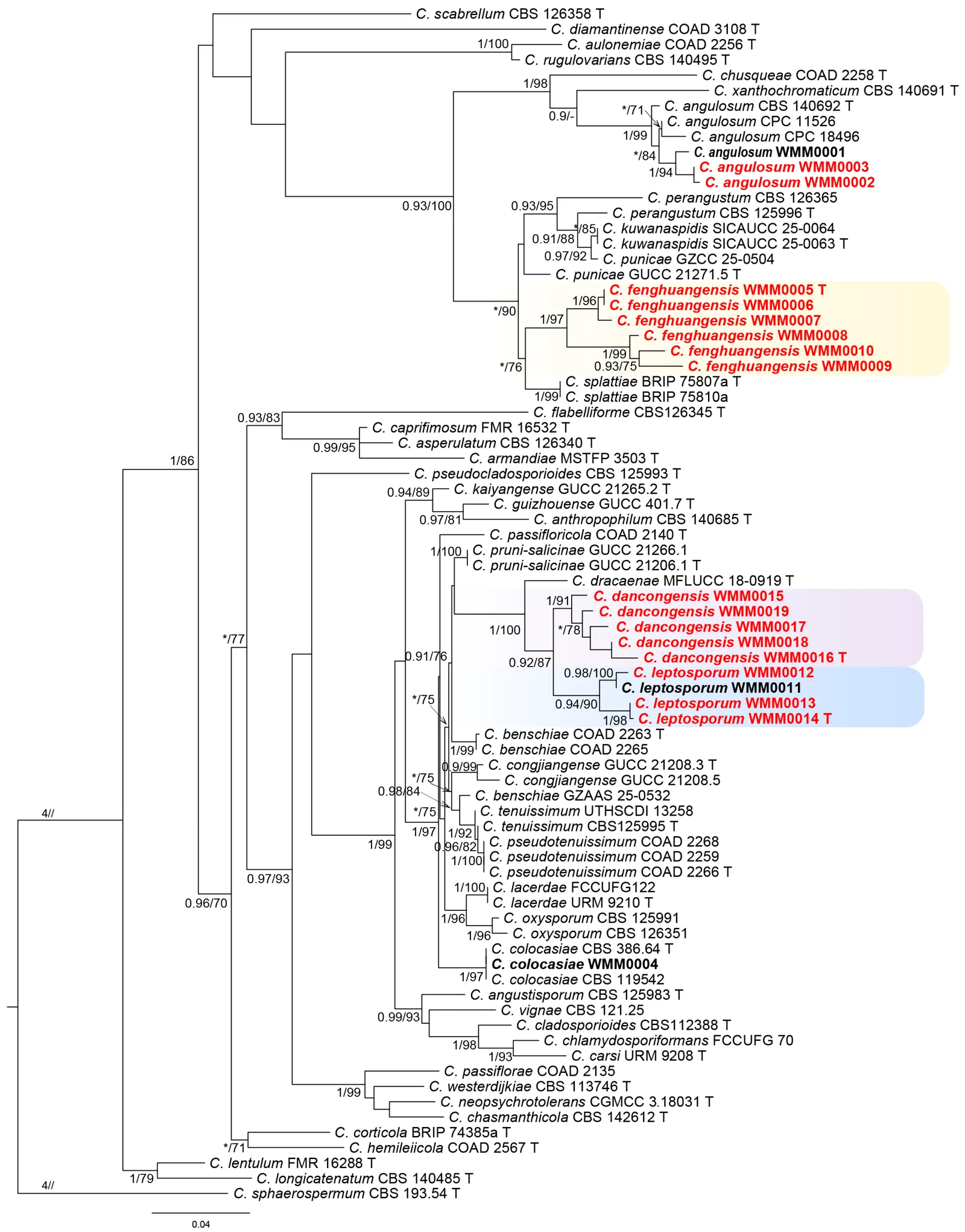
Fifty percent majority rule consensus tree from a Bayesian analysis based on a three-locus combined dataset (ITS-act-tef1-α) showing the phylogenetic relationships of the *C. cladosporioides* complex. The Bayesian posterior probabilities (PP > 0.9) and PhyML bootstrap support value (BS > 70%) are displayed at the nodes (PP/BS). Nodes with PP and BS values below 0.9 and 70% are displayed as “*” and “-”, respectively. “4//” in the branches means shortening the actual branches length by 4 times. The tree was rooted to *C. sphaerospermum* CBS 193.54 T. Ex-type cultures are indicated with “T”. New species introduced in this paper are marked in colour blocks. The newly isolates obtained in this study are marked in bold. Isolates retrieved by the direct isolation method were labelled as red and the tissue isolation method as black.

The primary identity of the examined isolates was assessed via megablast against the NCBI database. Sequences from closely related species and morphologically similar taxa were retrieved to construct the dataset. The reference sequences of the known species shown in Figure 1 are listed in Table 1, with those in Figure S1 listed in Table S1. For each locus, sequences were aligned using ClustalX 2.1 [40], and the alignments were manually adjusted where necessary. The best-fitting nucleotide-substitution modes according to the Akaike Information Criterion (AIC) were selected using mrmodeltest2 [41]. Alignments derived from this study were deposited in TreeBASE (submission ID 32724), and taxonomic novelties were deposited in Fungal Names (https://nmdc.cn/fungalnames/; accessed on 15 June 2026).

Phylogenetic analyses of the combined dataset were performed using Bayesian inference (BI) and maximum-likelihood (ML) methods. The BI analyses were conducted using MrBayes v. 3.2.1 following the protocol of Wang et al. [42], with the optimisation of each locus treated as a partition in combined analyses, based on the Markov Chain Monte Carlo (MCMC) approach [43]. All characters were equally weighted, and gaps were treated as missing data. The stationarity of the analyses was determined by examining the standard deviation of split frequencies (<0.01) and −ln likelihood plots in AWTY [44]. The ML analyses were conducted using PhyML v. 3.0 [45], with 1000 bootstrap replicates. The general time-reversible model was applied with an invariable gamma-distribution rate variation (*GTR* + *I* + *G*).

## 3. Results

### 3.1. Phylogenetic Analyses

Phylogenetic analyses of the entire *C. cladosporioides* complex were performed using a combined dataset of ITS (551 bp), act (232 bp) and tef1-α (349 bp), and the results are shown in Figure S1. For the BI analysis, the *K80* + *I* model was selected for the ITS locus, while the *HKY* + *G* and *GTR* + *I* + *G* models were used for the act and tef1-α loci, respectively. Phylogenetic analyses of the reduced *C. cladosporioides* complex were performed using a combined dataset of ITS (504 bp), act (215 bp) and tef1-α (269 bp), as illustrated in Figure 1. For the BI analysis, the *K80* + *I* model was selected for the ITS locus, while the *HKY* + *G* and *GTR* + *I* + *G* models were used for the act and tef1-α loci, respectively. The resulting phylogeny demonstrated that the three new species introduced in this study formed three well-supported clades, namely *C. dancongensis*, *C. fenghuangensis* and *C. leptosporum* (Figure 1). *Cladosporium dancongensis* and *C. leptosporum* are closely related to *C. benschiae*, *C. congjiangense*, *C. dracaenae*, *C. leptosporum*, *C. pruni-salininae*, *C. pseudotenuissimum* and *C. tenuissimum* (Figure 1). Meanwhile, *C. fenghuangensis* clustered closely to *C. kuwanaspidis*, *C. perangustum*, *C. punicae* and *C. splattiae* (Figure 1).

### 3.2. Taxonomy

**Figure 2:**
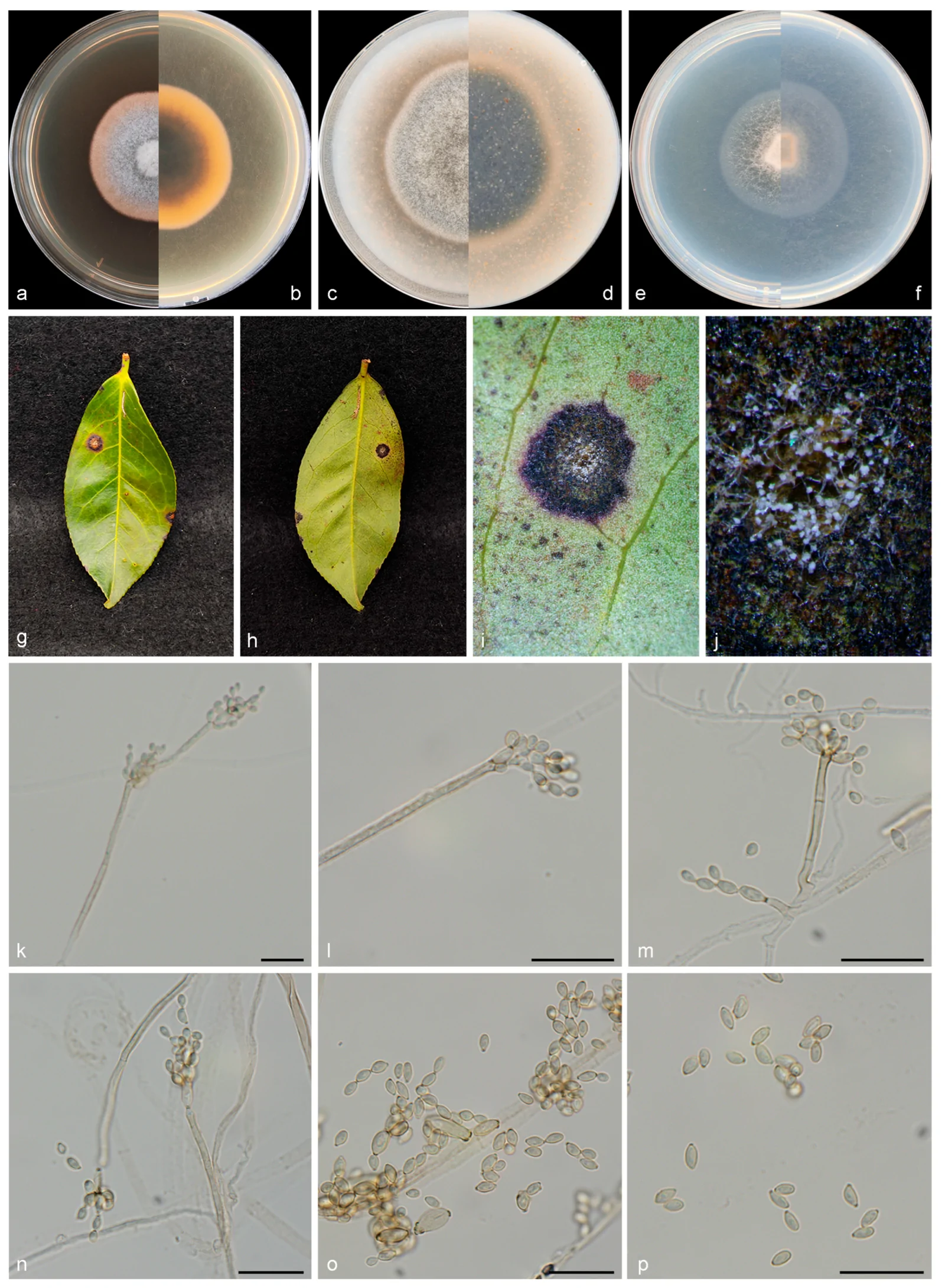
Separation site and morphological structure of *Cladosporium dancongensis* (holotype: WMM0016). (**a**–**f**) Colony morphology on MEA, OA, SNA media after 7 days of cultivation; (**g**,**h**) symptoms on the front and back of the leaf; (**i**,**j**). lesion morphology under a stereomicroscope; (**k**–**o**). morphological characteristics of conidiogenous structures; (**p**) morphological characteristics of conidia. Scale bars (**k**–**p**) = 10 μm.

***Cladosporium dancongensis*** M.M. Wang, M.Y. Sun & W. Li sp. nov. Figure 2.

FungalNames: FN 573808.

Etymology: The epithet is derived from the host plant, *Camellia sinensis* cv. *Fenghuang Dancong*.

Typus: CHINA, Guangdong Province, Chaozhou city, Fenghuang Mountain (23°53′20″ N 116°39′25″ E), from *Camellia sinensis* cv. *Fenghuang Dancong*, 8 January 2024, M.Y. Sun and Y.H. Tu (HMAS 354895, holotype designated here, dried culture on PDA; culture ex-type CGMCC 3.28721 = WMM0016).

Description: *Hyphae* variable in width, 1.0–3.3 μm wide ($\bar{x}$ = 1.98 μm; n = 20), subhyaline to pale brown, branched, septate. *Conidiophores* micronematous, solitary, arising terminally and laterally from hyphae, cylindrical–oblong to almost filiform, variable in width, 0.5–2.2 μm wide ($\bar{x}$ = 1.38 μm), septate, pale to medium brown or olivaceous-brown, becoming darker with age. *Conidiogenous cells* integrated, terminal and intercalary, cylindrical–oblong or moniliform, with 1–4 subdenticulate conidiogenous loci crowded at the apex, hyaline to pale olivaceous-brown, becoming darker with age. *Ramoconidia* pale olivaceous, occasionally formed, limoniform, apex truncate, 4.3–5.1 × 1.2–1.9 μm ($\bar{x}$ = 4.62 × 1.37 μm; n = 50), aseptate. *Conidia* pale olivaceous, catenate, ellipsoid, limoniform, subfusiform, with obovoid apex, 1.8–3.3 × 1.0–1.6 μm ($\bar{x}$ = 2.56 × 1.29 μm; n = 50), aseptate.

Culture characteristics (after 7 days at 25 °C): On PDA, colonies reaching 33–35 mm diam, surface oatmeal-coloured; reverse brownish-black; margin wheat-coloured, regular; aerial mycelia abundant, felt-like to velvety. On OA, colonies reaching 33–36 mm diam, brownish-grey to pale olivaceous-grey with white aerial mycelia; reverse brownish-black; margin wheat-coloured; aerial mycelia dense, lanose to felt-like. On SNA, colonies reaching 22–34 mm diam, surface pale white to subtransparent, reverse milky white; aerial mycelia sparse, felt-like. On MEA, colonies reaching 26–28 mm diam, greyish-white to pale olivaceous-grey; reverse brownish-black to yellow with ring patterns; aerial mycelia abundant, white.

Other examined isolates: CHINA, Guangdong Province, Chaozhou city, Fenghuang Mountain (23°55′39″ N 116°38′8″ E), from *Camellia sinensis* cv. *Fenghuang Dancong*, 8 January 2024, M.Y. Sun and Y.H. Tu (WMM0017, WMM0018, WMM0019); ibid. Fenghuang Mountain (23°53′20″ N 116°39′25″ E; WMM0015).

Notes: *Cladosporium dancongensis* was phylogenetically clustered together with *C. benschiae*, *C. congjiangense*, *C. dracaenae*, *C. leptosporum*, *C. pruni-salininae*, *C. pseudotenuissimum* and *C. tenuissimum* (Figure 1) but differs by 45 bp (7 bp in ITS, 21 bp in act, 17 bp in tef1-α), 33 bp (6 bp in ITS, 11 bp in act, 16 bp in tef1-α), 27 bp (3 bp in ITS, act absent, 24 bp in tef1-α), 31 bp (9 bp in ITS, 22 bp in act, tef1-α absent), 49 bp (9 bp in ITS, 21 bp in act, 19 bp in tef1-α), 42 bp (5 bp in ITS, 16 bp in act, 21 bp in tef1-α), and 46 bp (7 bp in ITS, 21 bp in act, 18 bp in tef1-α) in the ITS-act-tef1-α loci between the differences of type specimens, respectively. In terms of morphology, the eight species are distinguished by their distinct shapes, separation and size of conidia (e.g., conidia ellipsoid, limoniform, subfusiform, 1.8–3.3 × 1.0–1.6 μm, in *C. dancongensis*; vs. conidia subglobose, ellipsoid–ovoid, obovoid, fusiform, subcylindrical, 3.0–6.5 × 2.0–4.0 µm in *C. pruni-salicinae*, conidia globose, subglobose or ovoid, 1.3–3.3 × 1.1–1.8 μm in *C. leptosporum*, and small terminal conidia subglobose, obovoid, limoniform, sometimes globose, (2–)2.5–5(–6) × (1.5–)2–3 µm, intercalary conidia ovoid, ellipsoid or subcylindrical, 4–12(–17) × (1–)2–3(–4.5) µm in *C. tenuissimum*) [6,46]. The details of the morphological compositions are displayed in Table 2. This study proposes a novel classification of *C. dancongensis*, which is introduced as a new species, combining morphological characters and phylogenetic analyses.

**Figure 3:**
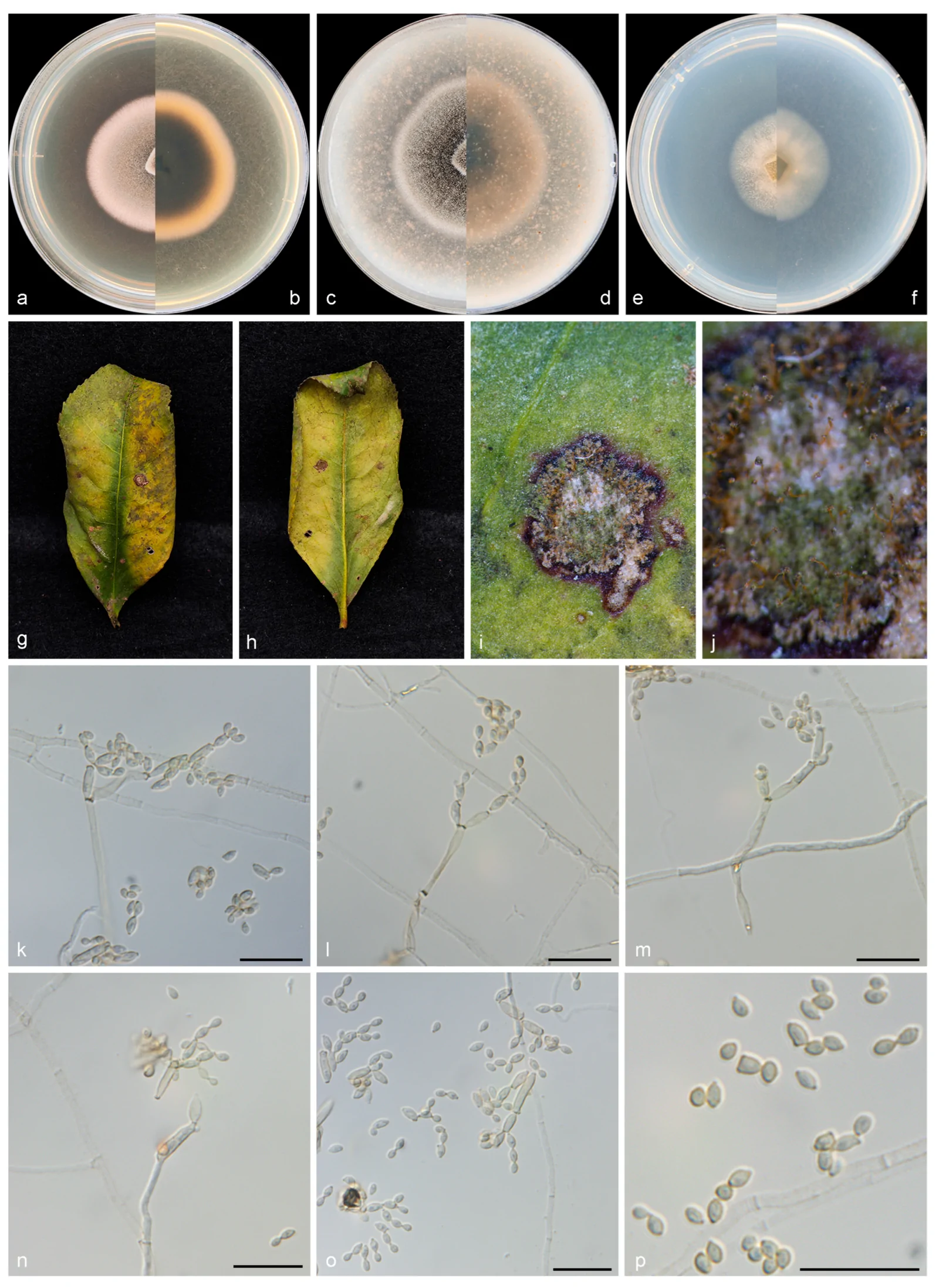
Separation site and morphological structure of *Cladosporium fenghuangensis* (holotype WMM0005). (**a**–**f**) Colony morphology on MEA, OA, SNA media after 7 days of cultivation; (**g**,**h**) symptoms on the front and back of the leaf; (**i**,**j**) lesion morphology under a stereomicroscope; (**k**–**o**) morphological characteristics of conidiogenous structures; (**p**) morphological characteristics of conidia. Scale bars (**k**–**p**) = 10 μm.

***Cladosporium fenghuangensis*** M.M. Wang, M.Y. Sun & W. Li sp. nov. Figure 3.

FungalNames: FN 573809.

Etymology: Epithet refers to the location of the type specimen, Fenghuang Mountain.

Typus: CHINA, Guangdong Province, Chaozhou city, Fenghuang Mountain (23°56′6″ N 116°40′38″ E), from *Camellia sinensis* cv. *Fenghuang Dancong*, 8 January 2024, M.Y. Sun and Y.H. Tu (HMAS 354896, holotype designated here, dried culture on PDA; culture ex-type CGMCC 3.28722 = WMM0005).

Description: *Hyphae* filiform to narrowly cylindrical, loosely branched, 0.4–1.8 μm wide ($\bar{x}$ = 1.15 μm), septate, subhyaline to pale olivaceous, walls almost unthickened. *Conidiophores* micronematous, solitary, arising terminally and laterally from hyphae, erect, straight or slightly flexuous, filiform to narrowly cylindrical-oblong, septate, usually not constricted at septa, occasionally septa darkened, pale olivaceous-brown, walls slightly thickened. *Conidiogenous cells* terminal, occasionally intercalary, narrowly cylindrical–oblong, occasionally septate, conidiogenous loci situated on small peg-like outgrowths, with 2–3 apically crowded loci, scars conspicuous, thickened and darkened, hyaline to pale grey–brown. *Ramoconidia* hyaline to pale grey–brown, fusiform to narrowly cylindrical, aseptate, with distinctly thickened and darkened scars at both ends, giving rise to branched conidial chains, 4.1–8.3 × 1.2–1.9 μm ($\bar{x}$ = 6.77 × 2.19 μm; n = 50), aseptate. *Conidia* hyaline to pale grey–brown, numerous, forming branched conidial chains with multidirectional branching; *terminal conidia* globose, subglobose or ovoid, apex broadly rounded, base with a single thickened scar, 1.2–2.4 × 0.9–1.4 μm ($\bar{x}$ = 1.76 × 1.14 μm; n = 50), aseptate; *intercalary conidia* hyaline to pale grey–brown, ovoid, limoniform to ellipsoid, somewhat fusiform, attenuated towards apex and base, with 1–3 distal hila, capable of further differentiating into secondary branched conidial chains, aseptate.

Culture characteristics (after 7 days at 25 °C): On PDA, colonies reaching 31–33 mm diam, olivaceous to olivaceous-grey; reverse olivaceous to olivaceous-black; felt-like, margin plumose, regular; aerial mycelia felt-like; spreading, sometimes with radial grooves; sporulation profuse. On OA, colonies reaching 30–33 mm diam, white to smoke-grey and pale olivaceous-grey; reverse pale olivaceous-grey to olive; velvety to floccose, margin white, regular; aerial mycelia abundant, dense, white to olivaceous-grey. On SNA, colonies reaching 20–22 mm diam, white (both sides); felt-like to velvety, margin pale white, centre pure white; aerial mycelia abundant; sporulating. On MEA, colonies reaching 26–29 mm diam, white to pale olivaceous-grey; reverse olive to brownish-black; felt-like to floccose; aerial mycelia abundant, white to pale grey.

Other examined isolates: CHINA, Guangdong Province, Chaozhou city, Fenghuang Mountain (23°56′6″ N 116°40′38″ E), from *Camellia sinensis* cv. *Fenghuang Dancong*, 8 January 2024, M.Y. Sun and Y.H. Tu (WMM0006, WMM0007, WMM0008, WMM0009, WMM0010).

Notes: Multi-locus phylogenetic analyses demonstrated that *C. fenghuangensis* constitutes a well-supported monophyletic lineage sister to *C. kuwanaspidis*, *C. perangustum*, *C. punicae* and *C. splattiae* (Figure 1). However, it differs by 5 to 9 bp in the ITS, 11 to 27 bp in the act, and 13 to 24 bp in the tef1-α loci among the type specimens of the aforementioned species, respectively. Morphologically, *C. fenghuangensis* differs from *C. kuwanaspidis* and *C. punicae* in the shape and size of conidia (subglobose or ovoid, 1.2–2.4 × 0.9–1.4 μm in *C. fenghuangensis* vs. ellipsoid–ovoid, obovoid, 2–7.5 × 2–4 μm in *C. kuwanaspidis*, and obovoid, ovoid, fusiform to ellipsoid, fusiform, subcylindrical, 2.5–5.5 × 2.0–3.5 µm in *C. punicae*) [25,49], and from *C. perangustum* in the size and separation of conidia (1.21–2.34 × 0.90–1.34 μm, aseptate in *C. fenghuangensis* vs. 2–16 × 2–3 µm, 0–1(–3)-septate in *C. perangustum*) [6]. Although there has been a lack of morphological description for the closest related species (*C. splattiae*) recently [50], the obvious molecular differences can still be regarded as evidence that these are two distinct species (7 bp in the ITS, 14 bp in the act and 13 bp in the tef1-α). As demonstrated in Table 2, a comprehensive array of morphological compositions is presented. Combining morphological characters and phylogenetic analyses, *C. fenghuangensis* was introduced as new species in this study.

**Figure 4:**
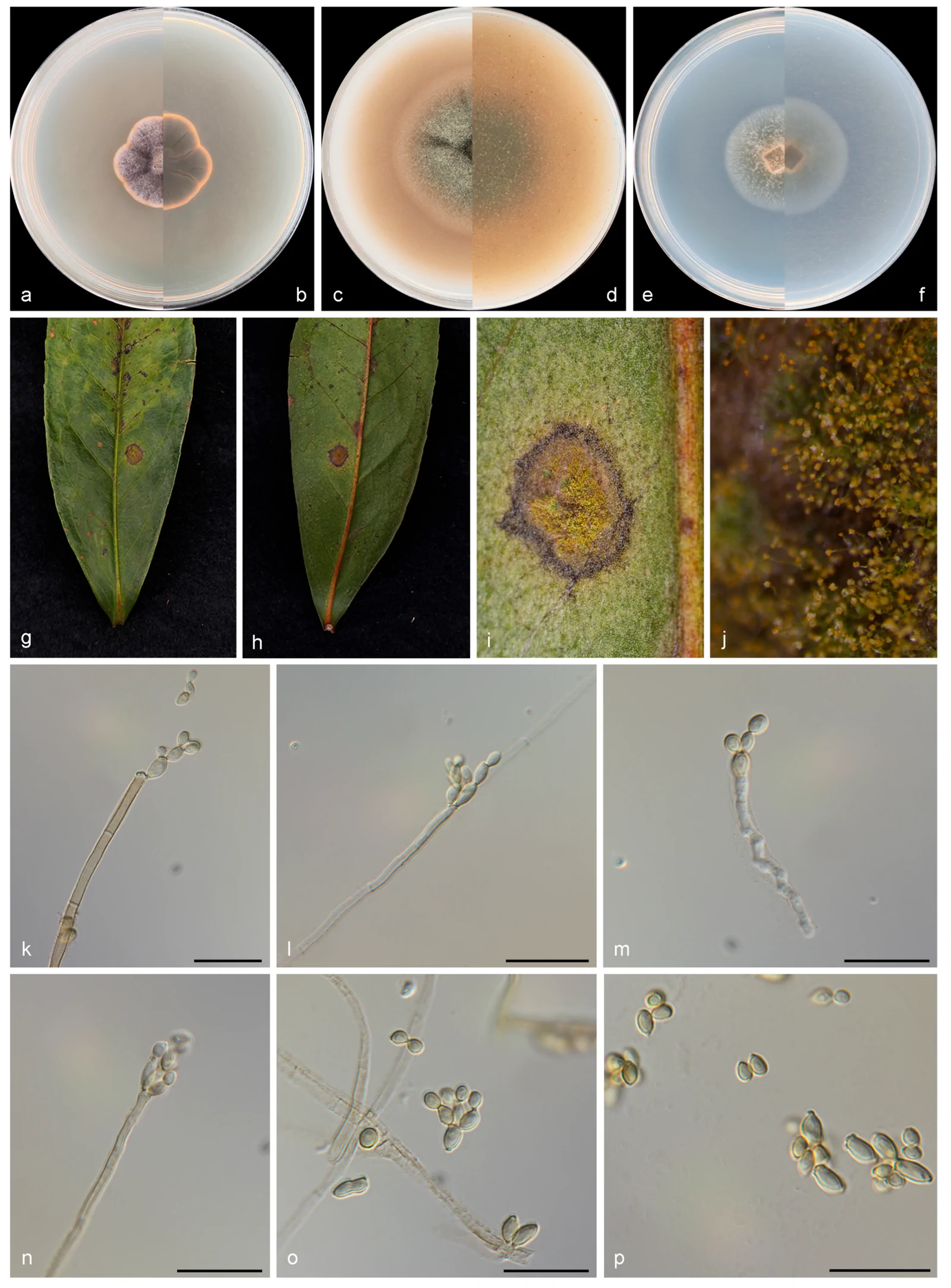
Separation site and morphological structure of *Cladosporium leptosporum* (holotype: WMM0014). (**a**–**f**) Colony morphology on MEA, OA, SNA media after 7 days of cultivation; (**g**,**h**) symptoms on the front and back of the leaf; (**i**,**j**). lesion morphology under a stereomicroscope; (**k**–**o**) morphological characteristics of conidiogenous structures; (**p**) morphological characteristics of conidia. Scale bars (**k**–**p**) = 10 μm.

***Cladosporium leptosporum*** M.M. Wang, M.Y. Sun & W. Li, sp. nov. Figure 4.

FungalNames: FN 573810.

Etymology: The epithet is derived from the combination of two Greek words leptos (narrow, slender) and spora (spore), referring to the narrow conidia.

Typus: CHINA, Guangdong Province, Chaozhou city, Fenghuang Mountain (23°53′8″ N 116°39′40″ E), from *Camellia sinensis* cv. *Fenghuang Dancong*, 8 July 2024, M.Y. Sun and Y.H. Tu (HMAS 354897, holotype designated here, dried culture on PDA; culture ex-type CGMCC 3.28723 = WMM0014).

Description: *Hyphae* 0.72–1.96 μm wide ($\bar{x}$ = 1.27 μm), pluriseptate, colourless to pale olivaceous. *Conidiophores* micronematous, solitary, arising terminally and laterally from hyphae, relatively long, almost unbranched, erect, straight or slightly flexuous, filiform to narrowly cylindrical–oblong, 0.7–2.0 μm wide ($\bar{x}$ = 1.31 μm), septate, pale brown to brown, walls slightly thickened. *Conidiogenous cells* mostly terminal, cylindrical, typically with 1–4 prominent apical conidiogenous loci, hyaline to pale brown, becoming darker with age. *Ramoconidia* pale brown, occasionally formed, subcylindrical to cylindrical, base or apex truncate, 3.3–4.2 × 1.4–1.9 μm ($\bar{x}$ = 3.77 × 1.24 μm; n = 50), aseptate. *Conidia* pale brown, numerous, acrogenous, sometimes catenate, aseptate, subhyaline or pale olivaceous; *intercalary conidia* pale brown, ellipsoid, limoniform or subfusiform; *terminal conidia* subglobose, measuring 1.3–3.3 × 1.1–1.8 μm ($\bar{x}$ = 2.07 × 1.42 μm; n = 50).

Culture characteristics (after 7 days at 25 °C): On PDA, colonies reaching 32–35 mm diam, olivaceous to olivaceous-grey; felt-like; spreading. On OA, colonies reaching 30–33 mm diam, dark brown, appearing olivaceous-black due to aerial mycelia, margin white; reverse dark brown with radial folds; aerial mycelia abundant, white, lanose, low. On SNA, colonies reaching 20–22 mm diam, milky white (both sides), felt-like; aerial mycelia sparse, white. On MEA, colonies reaching 17–20 mm diam, centre dark coffee-coloured, margin brownish-yellow; reverse brownish-black to yellow; aerial mycelia abundant, white or pale brown; with radial grooves on the surface.

Other examined isolates: CHINA, Guangdong Province, Chaozhou city, Fenghuang Mountain (23°55′39″ N 116°38′8″ E), from *Camellia sinensis* cv. *Fenghuang Dancong*, 8 January 2024, M.Y. Sun and Y.H. Tu (WMM0011, WMM0013); ibid. Fenghuang Mountain (23°53′20″ N 116°39′25″ E; WMM0013).

Notes: Phylogenetic analyses based on the combined ITS, act, and tef1-α dataset placed *C. leptosporum* within the *C. cladosporioides* complex. This finding was supported by a well-supported clade, which including *C. benschiae, C. congjiangense, C. dancongensis*, *C. dracaenae, C. leptosporum*, *C. pruni-salininae, C. pseudotenuissimum and C. tenuissimum* (Figure 1). The type specimen of the new species is clearly differentiated from the type specimens of these close relatives by substantial nucleotide differences. Specifically, *C. leptosporum* differs from *C. benschiae* by 29 bp (12 bp in ITS, 17 bp in act), from *C. congjiangense* by 27 bp (11 bp in ITS, 16 bp in act), from *C. dancongensis* by 31 bp (9 bp in ITS, 22 bp in act), from *C. dracaenae* by 9 bp (9 bp in ITS, act absent), from *C. pruni-salininae* by 31 bp (10 bp in ITS, 21 bp in act), from *C. pseudotenuissimum* by 29 bp (9 bp in ITS, 20 bp in act), and from *C. tenuissimum* by 33 bp (13 bp in ITS, 20 bp in act) when comparing sequences from the type specimens. As demonstrated in Table 2, detailed morphological comparisons have been conducted, which have indicated that this new species is distinguishable from its relatives in terms of shape, size, colour and septation of conidia. Combining morphological characters and phylogenetic analyses, *C. leptosporum* was introduced as new species in this study.

## 4. Discussion

The genus *Cladosporium* is famous for its notable species diversity [3] and its capacity to occupy a broad spectrum of ecological niches, encompassing both terrestrial and marine ecosystems [6,15,51]. Members of this genus are also well-known as plant pathogens, capable of causing a variety of symptoms such as leaf spot on *Glycine max* [52], panicle rot on *Oryza sativa* [53], and sooty spot on mandarin [54]. Nevertheless, the occurrence of these phenomena on *Camellia* spp. has not been elucidated until the present moment. Few studies have been published on the subject, reporting the presence of *Cladosporium* spp. on *Camellia sinensis* in China, South Korea and Japan, but no pathogenicity has been discovered [28,29,31,55].

In this study, nineteen *Cladosporium* isolates were obtained from symptomatic leaves of *Camellia sinensis* cv. *Fenghuang Dancong* (see Figure 1). These isolates were identified as belonging to three new species and two known species. It has been established that one of the known species *C. colocasiae* has previously been linked to leaf lesions in *Colocasia esculanta* [6]. Furthermore, closely related species of the three new species have also been reported as plant pathogens. For instance, *C. perangustum* causes leaf spot on *Syagrus oleracea* [56], and *C. tenuissimum* causes female cone disease on *Torreya grandis* [57] and has also been identified as a detrimental pathogen to citrus fruits [21]. It is important to note that pathogenicity tests were not conducted in the present study; therefore, no causal relationship can be established between the newly described *Cladosporium* species and the observed tea leaf spot symptoms. In order to ascertain whether these species act as primary pathogens, secondary invaders, or endophytes on tea plants, controlled inoculation experiments fulfilling Koch’s postulates are required as future work. Further studies are required to determine the specific role of these species in tea leaf spot disease, with a focus on their pathogenicity, genomics, and metabolism (metabolomics analysis). These urgent investigations will also provide insights into the mechanisms involved in the emergence of tea leaf spot disease and ultimately help to develop sustainable solutions. Another species of interest, *C. angulosum*, has been reported in the literature in relation to human bronchoalveolar lavage fluid [18], submerged leaf litter [58] and other fungi (*Austropuccinia psidii*) [59]. This suggests that this species may possess a broader ecological adaptability.

In the domain of taxonomy, the three novel *Cladosporium* species introduced in this study are classified within the *C. cladosporioides* complex (Figure 1 and Figure S1). The moderate support for the phylogenetic separation of *C. dancongensis* from *C. leptosporum* in the reduced phylogenetic analyses (Figure 1) compared to the entire phylogenetic analyses (Figure S1) may be partially attributed to the absence of tef1-α in *C. leptosporum*, given that the tef1-α provides substantial phylogenetic signal for species-level discrimination within the genus *Cladosporium*. Nonetheless, a morphological comparison of the key diagnostic features (e.g., shapes, separation and size of conidia; Table 2) can further facilitate the distinction between these two species. In a similar manner, the position of *C. fenghuangensis* in Figure S1 is more clearly resolved than the lower nodal support observed in Figure 1. This difference can be explained by the fact that Figure S1 only includes the type specimens with all three genes, while the majority of isolates of this species failed to have one or two gene loci successfully amplified. This highlights the importance of complete multi-locus datasets for robust phylogenetic inference in *Cladosporium* [6,16,18].

A comparison of the three novel species with the other known species in this complex reveals the presence of fundamental morphological traits: unbranched or rarely branched conidiophores, acropetal conidial chains, and smooth to rough conidia [6]. However, a significant divergence is observed in the size of their conidia, with *C. fenghuangensis* exhibiting conidia measuring 1.2–2.4 × 0.9–1.4 μm, contrasted by the larger conidia of *C. punicae*, which measure 2.5–5.5 × 2.0–3.5 µm. This pronounced miniature morphology is hypothesised to confer ecological advantages. As *Cladosporium* spp. are among the most prevalent airborne microorganisms [4], smaller spores enhance the dispersal efficiency and enable easier penetration into micro-niches such as stomata. Furthermore, their reduced size likely facilitates rapid colonisation as secondary invaders [14], while allowing for greater reproductive output under limited resources—a classic r-selection strategy.

Members in the *C. cladosporioides* species complex are globally recognised as the dominant *Cladosporium* lineage on plants, a pattern consistently observed across diverse crops and geographic regions. Surveys on *Citrus* spp. in China, fruit trees in Guizhou, tea plants, macadamia in Australia, and bamboo in Brazil have all demonstrated that this complex accounts for the vast majority of isolates, often exceeding 90% [21,25,47,54,60]. This ecological dominance is attributed to an expanded genomic repertoire of carbohydrate-active enzymes (CAZymes) and plant cell wall-degrading enzymes (PCWDEs), which facilitate the efficient colonisation of plant tissues [21]. While the majority of species within this complex are broad-spectrum saprobes or secondary invaders, some exhibit host restriction, for example, *C. vignae* on *Vigna* sp. and *C. cucumerinum* on *Cucurbitaceae* [4,6]. Geographically, species of this complex are cosmopolitan, but certain taxa show regional preferences; for example, *C. tenuissimum* is particularly common in tropical and subtropical regions [4].

The three new species described herein, isolated from *Camellia sinensis* cv. *Fenghuang Dancong* in China, conform to this established pattern. The placement of these organisms within the *C. cladosporioides* complex is consistent with the predominance of this group on plant hosts. The fact that these organisms were recovered from asymptomatic tissues suggests that they may function as endophytes or latent saprobes, a common lifestyle for members of this complex [6,14]. Their discovery on tea plants adds to the growing list of plant species harbouring novel *Cladosporium* diversity from China, a recognised hotspot for this genus [35,61,62]. It is recommended that subsequent studies examine the geographical distribution of these novel species across different tea-growing provinces in China and evaluate their host range. This will ascertain whether they are specific to *Camellia sinensis* or if they are more widely distributed on other plants.

## 5. Conclusions

In this study, three novel *Cladosporium* species isolated from *Camellia sinensis* cv. *Fenghuang Dancong* in China are formally described and illustrated. Based on a polyphasic approach integrating multi-locus phylogenetic analyses (ITS, act and tef1-α) and detailed morphological comparisons, the three taxa are assigned to the *C. cladosporioides* complex. They form distinct well-supported lineages and are clearly differentiated from their closest relatives (e.g., *C. congjiangense*, *C. tenuissimum*) by unique combinations of conidial dimensions and molecular characters [4,6,25]. This discovery adds to the growing body of knowledge on the high cryptic diversity within the *C. cladosporioides* complex, particularly from under-explored hosts and regions [16,35].

## Figures and Tables

**Table 1:** Information of substrate, country and GenBank accession number of species analysed in Figure 1 in this study.

Species	Strain	Substrate	Country	GenBank Accession No.		
				ITS	tef1-α	act
*C. angulosum*	CBS 140692 = UTHSC DI-13-235 = FMR 13348 T	Man	USA	LN834425	LN834521	LN834609
	CPC 11526	*Acacia mangium*	Thailand	HM148127	HM148371	HM148616
	CPC 18496	*Ananas comosus*	Panama	KT600414	KT600512	KT600610
	WMM0001	*Camellia sinensis*	China	PZ395190	PZ683653	PZ683639
	WMM0002	*Camellia sinensis*	China	PZ395191	PZ683654	PZ683640
	WMM0003	*Camellia sinensis*	China	PZ395192	—	PZ683641
*C. angustisporum*	CBS 125983 = CPC 12437 T	*Alloxylon wickhamii*	Australia	HM147995	HM148236	HM148482
*C. anthropophilum*	CBS 140685 = FMR 13382 = UTHSC DI-13-269 T	Man	USA	LN834437	LN834533	LN834621
*C. angustisporum*	CBS 125983 = CPC 12437 T	*Alloxylon wickhamii*	Australia	HM147995	HM148236	HM148482
*C. anthropophilum*	CBS 140685 = FMR 13382 = UTHSC DI-13-269 T	Man	USA	LN834437	LN834533	LN834621
*C. armandiae*	MST FP3503 T	Unidentified dead spider	Australia	PQ066521	PQ067355	PQ067354
*C. asperulatum*	CBS 126340 = CPC 14040 T	Protea susannae	Portugal	HM147998	HM148239	HM148485
*C. aulonemiae*	COAD 2256 T	*Aulonemia amplissima*	Brazil	MZ318427	MT680198	MT373119
*C. benschiae*	COAD 2263 T	*Aulonemia amplissima*	Brazil	MZ318436	MT680207	MT373128
	COAD 2265	*Aulonemia amplissima*	Brazil	MZ318437	MT680208	MT373129
	GZAAS 25-0532	*Dicranopteris pedata*	China	PV138694	PV177092	PV188078
*C. caprifimosum*	FMR 16532 T	Goat dung	Spain	LR813198	LR813210	LR813205
*C. carsi*	URM 9208 = FCCUFG 73 T	Air	Brazil	PZ121170	PZ157647	PZ157720
*C. chasmanthicola*	CBS 142612 T	*Chasmanthe aethiopica*	South Africa	KY646221	KY646227	KY646224
*C. chlamydosporiformans*	FCCUFG 70	Air	Brazil	PZ121169	PZ157646	PZ157718
*C. chusqueae*	COAD 2258 T	*Chusquea urelytra*	Brazil	MZ318430	MT680201	MT373122
*C. cladosporioides*	CBS 112388 = DTO 039-G6 T	Indoor air	Germany	HM148003	HM148244	HM148490
*C. colocasiae*	CBS 386.64 = ATCC 200944 = MUCL 10084 T	*Colocasia esculenta*	China	HM148067	HM148310	HM148555
	CBS 119542	*Colocasia esculenta*	Japan	HM148066	HM148309	HM148554
	WMM0004	*Camellia sinensis*	China	PZ395193	—	—
*C. congjiangense*	GUCC 21208.3 T	*Passiflora edulis*	China	OP852675	OP859042	OP863094
	GUCC 21208.5	*Passiflora edulis*	China	OP852676	OP859043	OP863095
*C. corticola*	BRIP 74385a T	*Melaleuca quinquenervia*	Australia	OP256851	OP288998	OP288997
***C. dancongensis***	**WMM0015**	***Camellia sinensis***	**China**	**PZ395204**	**PZ683657**	**PZ683648**
	**WMM0016 = CGMCC 3.28721 T**	***Camellia sinensis***	**China**	**PZ395205**	**PZ683658**	**PZ683649**
	**WMM0017**	***Camellia sinensis***	**China**	**PZ395206**	**PZ683659**	**PZ683650**
	**WMM0018**	***Camellia sinensis***	**China**	**PZ395207**	—	—
	**WMM0019**	***Camellia sinensis***	**China**	**PZ395208**	**PZ683660**	**PZ683651**
*C. diamantinense*	COAD 3108 T	Air	Brazil	ON062328	ON982817	ON141933
*C. dracaenae*	MFLUCC180919 T	*Dracaena* sp.	Thailand	OM908927	OP099913	—
***C. fenghuangensis***	**WMM0005 = CGMCC 3.28722 T**	***Camellia sinensis***	**China**	**PZ395194**	**PZ683655**	**PZ683642**
	**WMM0006**	***Camellia sinensis***	**China**	**PZ395195**	—	—
	**WMM0007**	***Camellia sinensis***	**China**	**PZ395196**	—	**PZ683643**
	**WMM0008**	***Camellia sinensis***	**China**	**PZ395197**	—	—
	**WMM0009**	***Camellia sinensis***	**China**	**PZ395198**	—	**PZ683644**
	**WMM0010**	***Camellia sinensis***	**China**	**PZ395199**	**PZ683656**	**PZ683645**
*C. flabelliforme*	CBS 126345 = CPC 14523 T	*Melaleuca cajuputi*	Australia	HM148092	HM148336	HM148581
*C. guizhouense*	GUCC 401.7 T	*Eucommia ulmoides*	China	OL579741	OL504965	OL519780
*C. hemileiicola*	COAD 2567 T	Uredinia of *Hemileia vastatrix* on leaves of *Coffea arabica*	Brazil	OP535376	OP676087	OP598128
*C. kaiyangense*	GUCC 21265.2 T	*Eriobotrya japonica*	China	OP852665	OP859045	OP863097
*C. kuwanaspidis*	SICAUCC 25-0063 T	*Kuwanaspis howardi*	CHINA	PV156489	PV153480	PV153468
	SICAUCC 25-0064	*Kuwanaspis howardi*	CHINA	PV156490	PV153481	PV153469
*C. lacerdae*	FCCUFG 122	Air	Brazil	PZ121173	PZ157650	PZ157723
*C. lacerdae*	URM 9210 = FCCUFG 72 T	Air	Brazil	PZ121172	PZ157649	PZ157722
*C. lentulum*	FMR 16288T	Unidentified leaf litter	Spain	LR813203	LR813215	LR813209
***C. leptosporum***	**WMM0011**	***Camellia sinensis***	**China**	**PZ395200**	—	**PZ683646**
	**WMM0012**	***Camellia sinensis***	**China**	**PZ395201**	—	—
	**WMM0013**	***Camellia sinensis***	**China**	**PZ395202**	—	—
	**WMM0014 = CGMCC 3.28723 T**	***Camellia sinensis***	**China**	**PZ395203**	—	**PZ683647**
*C. longicatenatum*	CBS 140485 = CPC 17189 T	Unknown plant	Australia	KT600403	KT600500	KT600598
*C. neopsychrotolerans*	CGMCC 3.18031 T	*Saussurea involucrata*, rhizosphere soil	China	KX938383	KX938400	KX938366
*C. oxysporum*	CBS 125991 = CPC 14371 = BA 1738	Soil	China	HM148118	HM148362	HM148607
	CBS 126351	Indoor air	Venezuela	HM148119	HM148363	HM148608
*C. passiflorae*	COAD 2135	*Passiflora edulis*	Brazil	MH682175	MH724943	MH729795
*C. passifloricola*	COAD 2140 T	*Passiflora edulis*	Brazil	—	MH724948	MH729800
*C. perangustum*	CBS 125996 = CPC 13815 T	*Cussonia* sp.	South Africa	HM148121	HM148365	HM148610
	CBS 126365	Chasmothecia of *Phyllactinia guttata* on leaves of *Corylus avellana*	USA	HM148123	HM148367	HM148612
*C. prunisalicinae*	GUCC 21206.1 T	*Prunus salicina*	China	OP852683	OP859041	OP863092
	GUCC 21266.1	*Prunus salicina*	China	OP852684		OP863093
*C. pseudocladosporioides*	CBS 125993 = CPC 14189 T	Outside air	The Netherlands	HM148158	HM148402	HM148647
*C. pseudotenuissimum*	COAD 2259	*Chusquea anelytroides*	Brazil	MZ318438	MT680209	MT373130
	COAD 2266 T	*Chusquea urelytra*	Brazil	MZ318439	MT680211	MT373132
	COAD 2268	*Chusquea urelytra*	Brazil	MZ318441	MT680213	MT373134
*C. punicae*	GUCC 21271.5 T	*Punica granatum*	China	OP852672	OP859056	OP863108
	GZCC 25-0504	*Dicranopteris pedata*	China	PV138690	PV177095	PV188074
*C. rugulovarians*	CBS 140495 T	Unidentified *Poaceae*	Brazil	KT600459	KT600558	KT600656
*C. scabrellum*	CBS 126358 = CPC 14976 = HJS 1031 T	*Ruscus hypoglossum*	Slovenia	HM148195	HM148440	HM148685
*C. sphaerospermum*	CBS 193.54 = ATCC 11289 = IMI 49637 T	Man	The Netherlands	DQ780343	EU570261	EU570269
*C. splattiae*	BRIP 75807a T	Unidentified dead spider	Australia	OR290120	OR335739	OR335734
	BRIP 75810a	Unidentified dead spider	Australia	OR290121	OR335740	OR335735
*C. tenuissimum*	CBS 125995 = CPC 14253 T	*Lagerstroemia* sp.	USA	HM148197	HM148442	HM148687
	UTHSC DI-13-258	Human	USA	LN834404	LN834500	LN834588
*C. vignae*	CBS 121.25 = ATCC 200933 = MUCL 10110	*Vigna unguiculata*	USA	HM148227	HM148473	HM148718
*C. westerdijkiae*	CBS 113746 T	Bing cherry fruits	USA	HM148061	HM148303	HM148548
*C. xanthochromaticum*	CBS 140691 = UTHSC DI-13-211 = FMR 13324 T	Man, bronchoalveolar lavage fluid	USA	LN834415	LN834511	LN834599

Notes: *C.* = *Cladosporium*; Ex-type strains are indicated with the “T”. The taxa obtained in this study are in bold. “—” indicates the absence of sequence data in GenBank.

**Table 2:** Morphological comparison of three new *Cladosporium* species reported in this study with allied species.

Species	Conidiophores	Conidiogenous Cells	Conidia Characters			
			Colour	Shape	Separation	Size
*Cladosporium benschiae* [47]	solitary, macronematous	integrated, terminal, cylindrical oblong or cylindrical	*SRc* brown, pale brown to subhyaline, *Cc* pale brown to brown	*SRc* oblong–ellipsoid, cylindrical, cylindrical–ovoid, *Cc* ellipsoid, limoniform, ovoid, subglobose, *Tc* obovoid, globose and subglobose	*SRc* 0–1 septate	*SRc* 5.80–13.56 × 2.65–3.84 µm, *Cc* 3.85–6.09 × 2.04–3.04 µm, *Tc* 3.01–4.82 × 2.08–2.96 µm
*C. congjiangense* [46]	solitary or in small loose groups	–	*Rc* medium to dark brown, *Cc* pale or medium olivaceous-brown	*Rc* oblong, oblong–ellipsoid, subcylindrical to cylindrical, *Cc* subglobose to obovoid, obovoid, limoniform or ellipsoid	aseptate	*Rc* 4.5–9.5 × 2.5–5.5 µm, *Cc* 2.5–5.5 × 1.5–4.0 µm
*C. dancongensis* [46]	micronematous, solitary	cylindrical–oblong or moniliform	pale olivaceous	*Rc* limoniform, *Cc* ellipsoid, limoniform, subfusiform	aseptate	*Rc* 4.3–5.1 × 1.2–1.9 μm, *Cc* 1.8–3.3 × 1.0–1.6 μm
*C. dracaenae* [48]	macronematous	cylindrical	hyaline to dark brown	*STc* globose or subglobose	–	12.5–16.5 × 2.5–3.0 µm
*C. leptosporum*	micronematous, solitary	cylindrical	pale brown	*Rc* subcylindrical to cylindrical, *Cc* ellipsoid, limoniform or subfusiform, subglobose	aseptate	*Rc* 3.3–4.2 × 1.4–1.9 μm, *Cc* 1.3–3.3 × 1.1–1.8 μm
*C. pruni-salicinae* [46]	solitary or in small loose groups	–	*Rc* pale to medium olivaceous-brown, *Cc* light grey to yellow	*Rc* subcylindrical to cylindrical, *Cc* subglobose, ellipsoid-ovoid, obovoid, fusiform, subcylindrical	aseptate	*Rc* 4.5–9.0 × 2.5–4.5 µm, *Cc* 3.0–6.5 × 2.0–4.0 µm
*C. pseudotenuissimum* [47]	solitary, macronematous	integrated, cylindrical or cylindrical	*Rc* and *SRc* pale brown to brown, *Cc* subhyaline, pale brown to dark brown	*Rc* oblong–ellipsoid, cylindrical, *SRc* ellipsoid, cylindrical–oblong, sometimes ovoid, *Cc* fusiform, ellipsoid, ellipsoid–ovoid, ellipsoid–cylindrical, subglobose, limoniform, oblong, oblong–ellipsoid, *Tc* ovoid to subglobose	*Rc* aseptate, *SRc* 0–1 septate. *Cc* aseptate, occasionally 1-septate	*Rc* 33.31–53.21 × 4.54–5.09 μm, *SRc* 7.05–20.27 × 2.77–5.86 μm, *Cc* 4.27–6.95 × 2.24–3.74 μm, *Tc* 3.09–4.90 × 1.96–3.59 μm
*C. tenuissimum* [6]	macronematous and micronematous, solitary	integrated, cylindrical–oblong	pale brown or pale olivaceous-brown	*Rc* subcylindrical or cylindrical–oblong, *SRc* ellipsoid, fusiform to subcylindrical or cylindrical, *STc* subglobose, obovoid, limoniform, sometimes globose, *Ic* ovoid, ellipsoid or subcylindrical	*Rc* 0(−1)-septate, *SRc* 0–1(–2)-septate, *STc* aseptate, *Ic* aseptate, occasionally 1-septate	*Rc* 22–41 × 3–4(–5) µm, *SRc* (6–)7–25(–31) × (2–)2.5–4(–5) µm, *STc* (2–)2.5–5(–6) × (1.5–)2–3 µm, *Ic* 4–12(–17) × (1–)2–3(–4.5) µm
*C. fenghuangensis*	micronematous, solitary	narrowly cylindrical–oblong	hyaline to pale grey–brown	*Rc* fusiform to narrowly cylindrical, *Cc* globose, subglobose or ovoid	aseptate	*Rc* 4.1–8.3 × 1.2–1.9 μm, *Cc* 1.2–2.4 × 0.9–1.4 μm
*C. kuwanaspidis* [49]	fasciculate, usually macronematous, cylindrical	cylindrical	*Rc* olive to brown, *SRc* pale brown, *Cc* olive to brown	*Rc* ellipsoidal to subcylindrical, *SRc* oblong, *Cc* ellipsoid–ovoid, obovoid, *Ic* limoniform, oval to ellipsoid, *Tc* globose to ellipsoid	*Rc* septate or aseptate, *SRc* 0–1 septate, *Cc* aseptate	*Rc* 6–13.5 × 3–5.5 μm, *SRc* 5.5–9 × 2.5–5 μm, *Cc* 2–7.5 × 2–4 μm, *Ic* 3.5–7.5 × 2.5–4 μm, *Tc* 2–4 × 2–3.5 μm.
*C. punicae* [46]	macro- and micronematous, solitary	–	*SRc* pale olivaceous brown, *Cc* subhyaline to pale olivaceous-brown	*Cc* obovoid, ovoid, fusiform to ellipsoid, fusiform, subcylindrical	aseptate	*SRc* 3.0–7.0 × 2.0–3.5 µm, *Cc* 2.5–5.5 × 2.0–3.5 µm
*C. perangustum* [6]	macronematous, semimacronematous or micronematous, solitary, sometimes in pairs	integrated, narrowly cylindrical–oblong	pale olivaceous-brown	*Rc* cylindrical–oblong, *SRc* narrowly ellipsoid to cylindrical–oblong, *STc* globose, subglobose or ovoid to obovoid, *Ic* ovoid, limoniform to ellipsoid	*Rc* aseptate, rarely 1(–2)-septate, *SRc* 0–1(–3)-septate, *Ic* 0(–1)-septate	*Rc* 25–45 × 2.5–3(–4.5) µm, *SRc* 6–30(–34) × 2–3(–3.5) µm, *STc* 2–4(–5) × (1.5–)2–2.5 µm, *Ic* 4–16(–19) × 2–3(–3.5) µm
*C. splattiae* [50]	–	–	–	–	–	–

Notes: *Cc* = conidia, *Ic* = intercalary conidia, *Rc* = ramoconidia, *SRc* = secondary ramoconidia, *STc* = small terminal conidia, *Tc* = terminal conidia.

## Data Availability

All sequences generated in this study were submitted to GenBank (https://www.ncbi.nlm.nih.gov, accessed on 19 June 2026). Additional requests can be directed at the corresponding author.

## Supplementary Materials

The following supporting information can be downloaded at https://www.mdpi.com/article/10.3390/jof12080598/s1. Figure S1: Fifty percent majority rule consensus tree from a Bayesian analysis based on a three-locus combined dataset (ITS-act-tef1-α) showing the phylogenetic relationships of the entire *C. cladosporioides* complex. Table S1: Taxa used in Figure S1 in this study, along with their corresponding GenBank accession numbers.
