# Supplementary material for: Morpho-Molecular Approach Reveals Three Novel Cladosporium Species Associated with Camellia sinensis cv. Fenghuang Dancong in China
Source: J Fungi (Basel). 2026 Aug 11;12(8):598. doi: 10.3390/jof12080598 (PMC13514916; doi:10.3390/jof12080598)
Supplement: Supplementary file 1 [file jof-12-00598-s001.zip › Supplementary Figure S1_Cladosporium phylogenetic tree.pdf]

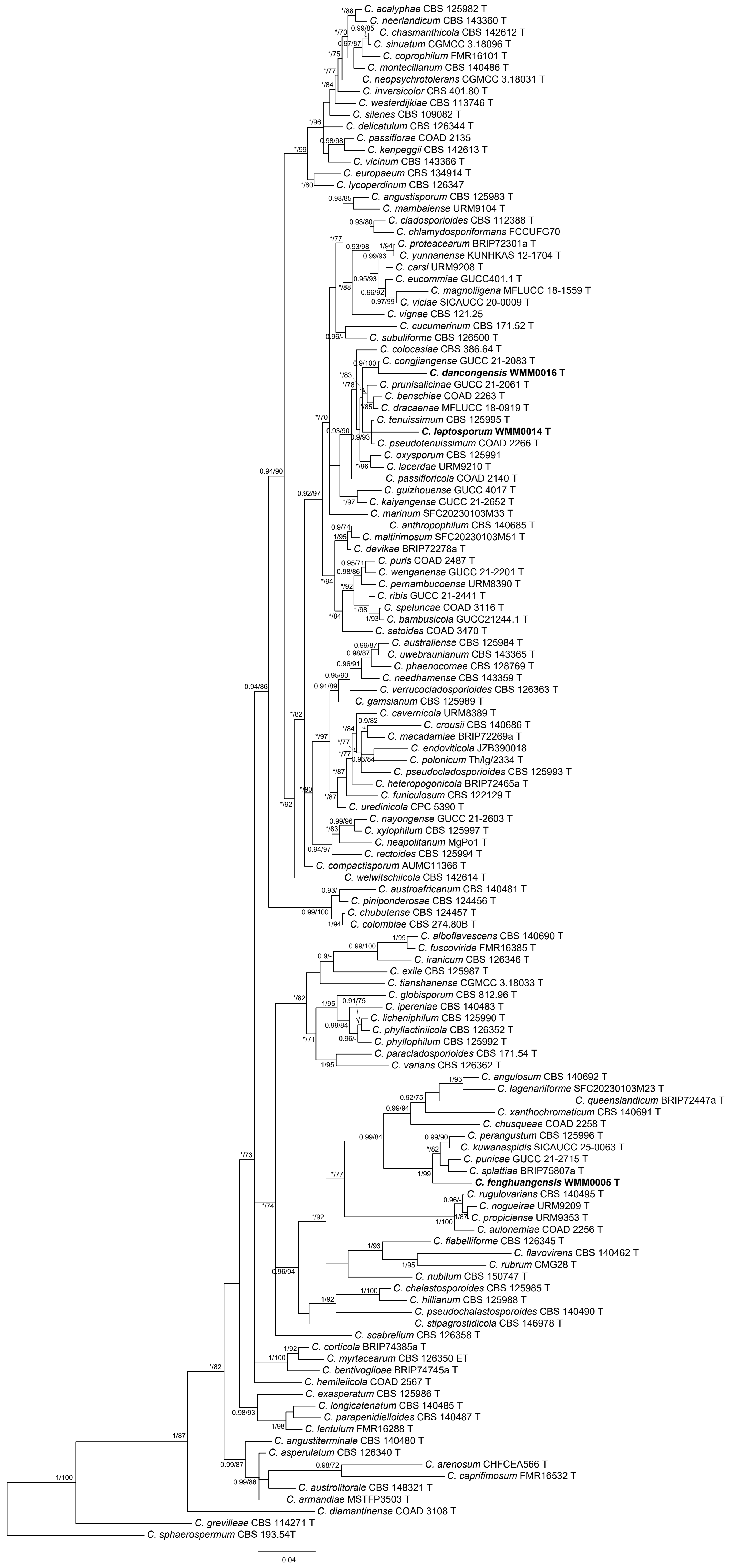
C. acalyphae CBS 125982 T
C. neerlandicum CBS 143360 T
C. chasmanthicola CBS 142612 T
C. sinuatum CGMCC 3.18096 T
C. coprophilum FMR16101 T
C. montecillanum CBS 140486 T
C. neopsychrotolerans CGMCC 3.18031 T
C. inversicolor CBS 401.80 T
C. westerdijkiae CBS 113746 T
C. silenes CBS 109082 T
C. delicatulum CBS 126344 T
C. passiflorae COAD 2135
C. kenpeggii CBS 142613 T
C. vicinum CBS 143366 T
C. europaeum CBS 134914 T
C. lycoperdinum CBS 126347
C. angustisporum CBS 125983 T
C. mambaiense URM9104 T
C. cladosporioides CBS 112388 T
C. chlamydosporiformans FCCUFG70
C. proteacearum BRIP72301a T
C. yunnanense KUNHKAS 12-1704 T
C. carsi URM9208 T
C. eucommiae GUCC401.1 T
C. magnoliigena MFLUCC 18-1559 T
C. viciae SICAUCC 20-0009 T
C. vignae CBS 121.25
C. cucumerinum CBS 171.52 T
C. subuliforme CBS 126500 T
C. colocasiae CBS 386.64 T
C. congjiangense GUCC 21-2083 T
C. dancongensis WMM0016 T
C. prunisalicinae GUCC 21-2061 T
C. benschiae COAD 2263 T
C. dracaenae MFLUCC 18-0919 T
C. tenuissimum CBS 125995 T
C. leptosporum WMM0014 T
C. pseudotenuissimum COAD 2266 T
C. oxysporum CBS 125991
C. lacerdae URM9210 T
C. passifloricola COAD 2140 T
C. guizhouense GUCC 4017 T
C. kaiyangense GUCC 21-2652 T
C. marinum SFC20230103M33 T
C. anthropophilum CBS 140685 T
C. maltirimosum SFC20230103M51 T
C. devikae BRIP72278a T
C. puris COAD 2487 T
C. wenganense GUCC 21-2201 T
C. pernambucoense URM8390 T
C. ribis GUCC 21-2441 T
C. speluncae COAD 3116 T
C. bambusicola GUCC21244.1 T
C. setoides COAD 3470 T
C. australiense CBS 125984 T
C. uwebraunianum CBS 143365 T
C. phaenocomae CBS 128769 T
C. needhamense CBS 143359 T
C. verrucocladosporioides CBS 126363 T
C. gamsianum CBS 125989 T
C. cavernicola URM8389 T
C. crousii CBS 140686 T
C. macadamiae BRIP72269a T
C. endoviticola JZB390018
C. polonicum Th/lg/2334 T
C. pseudocladosporioides CBS 125993 T
C. heteropogonicola BRIP72465a T
C. funiculosum CBS 122129 T
C. uredinicola CPC 5390 T
C. nayongense GUCC 21-2603 T
C. xylophilum CBS 125997 T
C. neapolitanum MgPo1 T
C. rectoides CBS 125994 T
C. compactisporum AUMC11366 T
C. welwitschiicola CBS 142614 T
C. austroafricanum CBS 140481 T
C. piniponderosae CBS 124456 T
C. chubutense CBS 124457 T
C. colombiae CBS 274.80B T
C. alboflavescens CBS 140690 T
C. fuscoviride FMR16385 T
C. iranicum CBS 126346 T
C. exile CBS 125987 T
C. tianshanense CGMCC 3.18033 T
C. globisporum CBS 812.96 T
C. ipereniae CBS 140483 T
C. licheniphilum CBS 125990 T
C. phyllactiniicola CBS 126352 T
C. phyllophilum CBS 125992 T
C. paracladosporioides CBS 171.54 T
C. varians CBS 126362 T
C. angulosum CBS 140692 T
C. lagenariiforme SFC20230103M23 T
C. queenslandicum BRIP72447a T
C. xanthochromaticum CBS 140691 T
C. chusqueae COAD 2258 T
C. perangustum CBS 125996 T
C. kuwanaspidis SICAUCC 25-0063 T
C. punicae GUCC 21-2715 T
C. splattiae BRIP75807a T
C. fenghuangensis WMM0005 T
C. rugulovarians CBS 140495 T
C. nogueirae URM9209 T
C. propiciense URM9353 T
C. aulonemiae COAD 2256 T
C. flabelliforme CBS 126345 T
C. flavovirens CBS 140462 T
C. rubrum CMG28 T
C. nubilum CBS 150747 T
C. chalastosporoides CBS 125985 T
C. hillianum CBS 125988 T
C. pseudochalastosporoides CBS 140490 T
C. stipagrostidicola CBS 146978 T
C. scabrellum CBS 126358 T
C. corticola BRIP74385a T
C. myrtacearum CBS 126350 ET
C. bentivoglioae BRIP74745a T
C. hemileiicola COAD 2567 T
C. exasperatum CBS 125986 T
C. longicatenatum CBS 140485 T
C. parapenidielloides CBS 140487 T
C. lentulum FMR16288 T
C. angustiterminale CBS 140480 T
C. asperulatum CBS 126340 T
C. arenosum CHFCEA566 T
C. caprifimosum FMR16532 T
C. australitorale CBS 148321 T
C. armandiae MSTFP3503 T
C. diamantinense COAD 3108 T
C. grevilleae CBS 114271 T
C. sphaerospermum CBS 193.54T
0.04
