# Supplementary material for: Morpho-Molecular Approach Reveals Three Novel Cladosporium Species Associated with Camellia sinensis cv. Fenghuang Dancong in China
Source: J Fungi (Basel). 2026 Aug 11;12(8):598. doi: 10.3390/jof12080598 (PMC13514916; doi:10.3390/jof12080598)
Supplement: Supplementary file 1 [file jof-12-00598-s001.zip › Table S1.pdf]

Table S1. Taxa used in Figure S1 in this study, along with their corresponding GenBank accession numbers.

Species	Strain	Substrate	Country	GenBank accession no.		
				ITS	tef1-α	act
C. acalyphae	CBS 125982 = CPC 11625 T	*Acalypha australis*	South Korea	HM147994	HM148235	HM148481
C. alboflavescens	CBS 140690 = UTHSC DI-13-225 = FMR 13338 T	Animal, bronchoalveolar lavage fluid	USA	LN834420	LN834516	LN834604
C. angulosum	CBS 140692 = UTHSC DI-13-235 = FMR 13348 T	Man	USA	LN834425	LN834521	LN834609
C. angustisporum	CBS 125983 = CPC 12437 T	*Alloxylon wickhamii*	Australia	HM147995	HM148236	HM148482
C. angustiterminale	CBS 140480 = CPC 15564 T	*Banksia grandis*	Australia	KT600379	KT600476	KT600575
C. anthropophilum	CBS 140685 = FMR 13382 = UTHSC DI-13-269 T	Man	USA	LN834437	LN834533	LN834621
C. arenosum	CHFC-EA 566 T	marine sediment sand	Antarctica	MN879328	MN890011	MN890008
C. armandiae	MST FP3503 T	an unidentified dead spider	Australia	PQ066521	PQ067355	PQ067354
C. asperulatum	CBS 126340 = CPC 14040 T	*Protea susannae*	Portugal	HM147998	HM148239	HM148485
C. aulonemiae	COAD 2256 T	*Aulonemia amplissima*	Brazil	MZ318427	MT680198	MT373119
C. australiense	CBS 125984 = CPC 13226 T	*Eucalyptus moluccana*	Australia	HM147999	HM148240	HM148486
C. austroafricanum	CBS 140481 = CPC 16763 T	Leaf litter	South Africa	KT600381	KT600478	KT600577
C. austrolitorale	CBS 148321 T	coastal sea sand	Antarctica	MN879327	MN890010	MN890007
C. bambusicola	GUCC 21244.1 ET	*Ribes burejense*	China	MZ318433	MT680204	MT373125
C. benschiae	COAD 2263T	*Aulonemia amplissima*	Brazil	MZ318436	MT680207	MT373128
C. bentivoglioae	BRIP 74745a T	Sporobolus creber (*Poaceae*)	Australia	OR947065	OR964961	OR964960
C. caprifimosum	FMR 16532 T	goat dung	Spain	LR813198	LR813210	LR813205
C. carsi	URM 9208 = FCCUFG 73 T	Air	Brazil	PZ121170	PZ157647	PZ157720
C. cavernicola	URM 8389 T	Air	Brazil	MZ518829	MZ555733	MZ555746
C. chalastosporoides	CBS 125985 = CPC 13864 T	Fruiting bodies of *Teratosphaeria proteaearboreae* on leaves of *Protea nitida*	South Africa	HM148001	HM148242	HM148488
C. chasmanthicola	CBS 142612 T	*Chasmanthe aethiopica*	South Africa	KY646221	KY646227	KY646224
C. chlamydosporiformans	FCCUFG 70	Air	Brazil	PZ121169	PZ157646	PZ157718
C. chubutense	CBS 124457 = CPC 13979 = CIEFAP 321 T	*Pinus ponderosa*	Argentina	FJ936158	FJ936161	FJ936165
C. chusqueae	COAD 2258 T	*Chusquea urelytra*	Brazil	MZ318430	MT680201	MT373122
C. cladosporioides	CBS 112388 = DTO 039-G6 T	Air	Germany	HM148003	HM148244	HM148490
C. colocasiae	CBS 386.64 = ATCC 200944 = MUCL 10084 T	*Colocasia esculenta*	China	HM148067	HM148310	HM148555
C. colombiae	CBS274.80B T	*Cortaderia* sp.	Colombia	FJ936159	FJ936163	FJ936166
C. compactisporum	AUMC 11366 T	Air	Egypt	MN826822	—	OL514010
C. congjiangense	GUCC 21208.3 T	*Passiflora edulis*	China	OP852675	OP859042	OP863094
C. coprophilum	FMR 16101 T	unidentified herbivore dung	Spain	LR813199	LR813211	LR813204
C. corticola	BRIP 74385a T	*Melaleuca quinquenervia*	Australia	OP256851	OP288998	OP288997

C. crousii	CBS 140686 = UTHSC DI-13-247 = FMR 13360 T	Man	USA	LN834431	LN834527	LN834615
C. cucumerinum	CBS 171.52 = MUCL 10092 T	*Cucumis sativus*	Netherlands	HM148072	HM148316	HM148561
C. delicatulum	CBS 126344 = CPC 11389 T	*Tilia cordata*	Germany	HM148081	HM148325	HM148570
C. devikae	BRIP 72278a T	*Macadamia integrifolia*	Australia	MZ303808	MZ344193	MZ344212
C. diamantinense	COAD 3108 T	air	Brazil	ON062328	ON982817	ON141933
C. dracaenae	MFLUCC180919 T	*Dracaena* sp.	Thailand	OM908927	OP099913	—
C. endoviticola	JZB390018 T	grapevine flowers	China	MN654960	MN984228	MN984220
C. eucommiae	GUCC 401.1 T	*Eucommia ulmoides*	China	OL587465	OL504966	OL519775
C. europaeum	CBS 134914 = CPC 14296 = BA1695 T	Indoor building material, school	Denmark	HM148056	HM148298	HM148543
C. exasperatum	CBS 125986 = CPC 1463 T	*Eucalyptus tintinnans*	Australia	HM148090	HM148334	HM148579
C. exile	CBS 125987 = CPC 11828 T	Chasmothecia of *Phyllactinia guttata* on leaves of *Corylus avellana*	USA	HM148091	HM148335	HM148580
C. flabelliforme	CBS 126345 = CPC 14523 T	*Melaleuca cajuputi*	Australia	HM148092	HM148336	HM148581
C. flavovirens	CBS 140462 = FMR 13386 = UTHSC DI-13-273 T	Man	USA	LN834440	LN834536	LN834624
C. funiculosum	CBS 122129 = ATCC 38010 = IFO 6537 = JCM 10683 = NBRC 6537 T	*Vigna umbellata*	Japan	HM148094	HM148338	HM148583
C. fuscoviride	FMR 16385 T	Garden soil	Spain	LR813200	LR813212	LR813206
C. gamsianum	CBS 125989 = CPC 11807 T	Strelitzia sp.	South Africa	HM148095	HM148339	HM148584
C. globisporum	CBS 812.96 T	Meat stamp	Sweden	HM148096	HM148340	HM148585
C. grevilleae	CBS 114271 = CPC 2913 = JT 974 T	*Grevillea* sp.	Australia	JF770450	JF770472	JF770473
C. guizhouense	GUCC 401.7 T	*Eucommia ulmoides*	China	OL579741	OL504965	OL519780
C. hemileiicola	COAD 2567 T	On uredinia of *Hemileia vastatrix* on leaves of *Coffea arabica*	Brazil	OP535376	OP676087	OP598128
C. heteropogonicola	BRIP 72465a T	*Heteropogon* sp. (*Poaceae*)	Australia	OL307932	OL332742	OL332743
C. hillianum	CBS 125988 = CPC 15459 = C92 T	*Typha orientalis*	New Zealand	HM148097	HM148341	HM148586
C. inversicolor	CBS 401.80 = ATCC 200941 T	*Triticum aestivum*	Netherlands	HM148101	HM148345	HM148590
C. ipereniae	CBS 140483 = CPC 16238 T	*Puya* sp.	Chile	KT600394	KT600491	KT600589
C. iranicum	CBS 126346 = CPC 11554 T	*Citrus sinensis*	Iran	HM148110	HM148354	HM148599
C. kaiyangense	GUCC 21265.2 T	*Eriobotrya japonica*	China	OP852665	OP859045	OP863097
C. kenpeggii	CBS 142613 T	*Passiflora edulis*	Australia	KY646222	KY646228	KY646225
C. kuwanaspidis	SICAUCC 25-0063 T	*Kuwanaspis howard*	China	PV156489	PV153480	PV153468
C. lacerdae	URM 9210 = FCCUFG 72 T	Air	Brazil	PZ121172	PZ157649	PZ157722
C. lagenariiforme	SFC20230103-M23 T	seaweed	Republic of Korea	OQ186119	OQ185128	OQ185167
C. lentulum	FMR 16288 T	unidentified leaf litter	Spain	LR813203	LR813215	LR813209

C. licheniphilum	CBS 125990 = CPC 13224 T	Lichen *Phaeophysica orbicularis* and Physcia sp. on stems and bark of *Acer platanoides*	Germany	HM148111	HM148355	HM148600
C. longicatenatum	CBS 140485 = CPC 17189 T	Unknown plant	Australia	KT600403	KT600500	KT600598
C. lycoperdinum	CBS 126347 = CPC 12102	Galls of *Apiosporina morbosa* on *Prunus* sp.	Canada	HM148112	HM148356	HM148601
C. macadamiae	BRIP 72269a T	*Macadamia integrifolia*	Australia	MZ303810	MZ344195	MZ344214
C. magnoliigena	MFLUCC 18-1559 T	*Magnolia grandiflora*	China	MK347813	MK340864	—
C. maltirimosum	SFC20230103-M51 T	Sea sand	South Korea	OQ186148	OQ185156	OQ185196
C. mambaiense	URM 9104 = FCCUFG 71 T	Air	Brazil	PZ121174	PZ157651	PZ157724
C. marinum	SFC20230103-M33 T	Seaweed	South Korea	OQ186129	OQ185137	OQ185177
C. montecillanum	CBS 140486 = CPC 17953 T	Pine needles	Mexico	KT600406	KT600504	KT600602
C. myrtacearum	CBS 126350 = CPC 14567 ET	*Corymbia foelscheana*	Australia	HM148117	HM148361	HM148606
C. nayongense	GUCC 21260.3 T	*Prunus pseudocerasus*	China	OP852669	OP859054	OP863106
C. neapolitanum	MgPo1 T	*Micromeria graeca*	Italy	MK387890	MK416094	MK416051
C. needhamense	CBS 143359 = CPC 22353 = EMSL 1866 T	Indoor air	USA	MF473142	MF473570	MF473991
C. neerlandicum	CBS 143360 = DTO 086-C5 T	Swab sample	Netherlands	KP701887	KP701764	KP702010
C. neopsychrotolerans	CGMCC 3.18031 T	*Saussurea involucrata*	China	KX938383	KX938400	KX938366
C. nogueirae	URM 9209 = FCCUFG 61 T	Air	Brazil	PZ121181	PZ157658	PZ157731
C. nubilum	CBS 150747 T	hypersaline brine	Poland	OR539697	OR557575	OR557574
C. oxysporum	CBS 125991 = CPC 14371 = BA 1738	Soil	China	HM148118	HM148362	HM148607
C. paracladosporioides	CBS 171.54 = ATCC 11278,200943 = IFO 6369 = IMI 049626 = MUCL 917 = NCTC 4097 T	-	-	HM148120	HM148364	HM148609
C. parapenidielloides	CBS 140487 = CPC 17193 T	*Eucalyptus* sp.	Australia	KT600410	KT600508	KT600606
C. passiflorae	COAD 2135	*Passiflora edulis*	Brazil	MH682175	MH724943	MH729795
C. passifloricola	COAD 2140 T	*Passiflora edulis*	Brazil	—	MH724948	MH729800
C. perangustum	CBS 125996 = CPC 13815 T	*Cussonia* sp.	South Africa	HM148121	HM148365	HM148610
C. pernambucoense	URM 8390 T	Air	Brazil	MZ518828	MZ555732	MZ555745
C. phaenocomae	CBS 128769 = CPC 18223 T	*Phaenocoma prolifera*	South Africa	JF499837	JF499875	JF499881
C. phyllactiniicola	CBS 126352 = CPC 11836 T	Chasmothecia of *Phyllactinia guttata* on leaves of *Corylus avellana*	USA	HM148150	HM148394	HM148639
C. phyllophilum	CBS 125992 = CPC 11333 T	*Taphrina* sp. on *Prunus cerasus*	Germany	HM148154	HM148398	HM148643
C. pini-ponderosae	CBS 124456 = CPC 13980 = CIEFAP 322 T	*Pinus ponderosa*	Argentina	FJ936160	FJ936164	FJ936167
C. polonicum	Th/lg/2334 T	From gall of *Asphondylia serpylli* on *Thymus vulgaris*	Poland	MK387894	MK416098	MK416055
C. propiciense	URM 9353 = FCCUFG 76 T	Air	Brazil	PZ121182	PZ157659	PZ157734

C. proteacearum	BRIP 72301a T	*Macadamia integrifolia*	Australia	MZ303809	MZ344194	MZ344213
C. prunisalicinae	GUCC 21206.1 T	*Prunus salicina*	China	OP852683	OP859041	OP863092
C. pseudochalastosporoides	CBS 140490 = CPC 17823 T	Pine needles	Mexico	KT600415	KT600513	KT600611
C. pseudocladosporioides	CBS 125993 = CPC 14189 T	Outside air	Netherlands	HM148158	HM148402	HM148647
C. pseudotenuissimum	COAD 2266 T	*Chusquea urelytra*	Brazil	MZ318439	MT680211	MT373132
C. punicae	GUCC 21271.5 T	*Punica granatum*	China	OP852672	OP859056	OP863108
C. puris	COAD 2487 T	submerged litter in streams	Brazil	MK253337	MK293777	MK249980
C. queenslandicum	BRIP 72447a T	*Arundinella nepalensis* (*Poaceae*)	Australia	OL307928	OL332735	OL332736
C. rectoides	CBS 125994 = CPC 11624 T	*Vitis flexuosa*	South Korea	HM148193	HM148438	HM148683
C. ribis	GUCC 21244.1 T	*Ribes burejense*	China	OP852666	OP859046	OP863098
C. rubrum	CMG 28 = MUM 19.39 T	*Vitis vinifera*	Portugal	MN053018	MN066644	MN066639
C. rugulovarians	CBS 140495 T	unidentified *Poaceae*	Brazil	KT600459	KT600558	KT600656
C. scabrellum	CBS 126358 = CPC 14976 = HJS 1031 T	*Ruscus hypoglossum*	Slovenia	HM148195	HM148440	HM148685
C. setoides	COAD 3470 T	On uredinia of *Hemileia vastatrix* on leaves of *Coffea arabica*	Ethiopia	OP535379	OP676090	OP598131
C. silenes	CBS 109082 T	*Silene maritima*	UK	EF679354	EF679429	EF679506
C. sinuatum	CGMCC 3.18096 T	Soil	China	KX938385	KX938402	KX938368
C. speluncae	COAD 3116 T	soil	Brazil	ON062329	ON982818	ON141934
C. splattiae	BRIP 75807a T	unidentified dead spider	Australia	OR290120	OR335739	OR335734
C. stipagrostidicola	CBS 146978 T	*Stipagrostis* cf. *ciliata*	Namibia	MZ064420	MZ078223	MZ078146
C. subuliforme	CBS 126500 = CPC 13735 T	*Chamaedorea metallica*	Thailand	HM148196	HM148441	HM148686
C. tenuissimum	CBS 125995 = CPC 14253 T	*Lagerstroemia* sp.	USA	HM148197	HM148442	HM148687
C. tianshanense	CGMCC 3.18033 T	*Saussurea involucrata,* rhizosphere soil	China	KX938381	KX938398	KX938364
C. uredinicola	CPC 5390 = ATCC 46649 T	Hyperparasite on *Cronartium fusiforme* f. sp. *quercum* on *Quercus nigra leaves*	USA	AY251071	HM148467	HM148712
C. uwebraunianum	CBS 143365 = DTO 072-D8 T	Indoor air	Netherlands	MF473306	MF473729	MF474156
C. varians	CBS 126362 = CPC 13658 = HAL 2061 F T	*Catalpa bungei*	Russia	HM148224	HM148470	HM148715
C. verrucocladosporioides	CBS 126363 = CPC 12300 T	*Rhus chinensis*	South Korea	HM148226	HM148472	HM148717
C. viciae	SICAUCC 20-0009 T	*Vicia faba*	China	PP736376	PP779577	PP782109
C. vicinum	CBS 143366 = CPC 22316 = EMSL 1819 T	Indoor air	USA	MF473311	MF473734	MF474161
C. vignae	CBS 121.25 = ATCC 200933 = MUCL 10110 T	*Vigna unguiculata*	USA	HM148227	HM148473	HM148718
C. welwitschiicola	CBS 142614 T	*Welwitschia mirabilis*	Namibia	KY646223	KY646229	KY646226
C. wenganense	GUCC 21220.1 T	*Prunus persica*	China	OP852682	OP859049	OP863101
C. westerdijkiae	CBS 113746 T	Bing cherry fruits	USA	HM148061	HM148303	HM148548

C. xanthochromaticum	CBS 140691 = UTHSC DI-13-211 = FMR 13324 T	Man	USA	LN834415	LN834511	LN834599
C. xylophilum	CBS 125997 = CPC 12403 T	*Picea abies*	Russia	HM148230	HM148476	HM148721
C. yunnanense	KUN HKAS 121704 T	*Paris polyphylla*	China	OK338502	OL825680	OL466937
C. sphaerospermum	CBS 193.54 = ATCC 11289 = IMI 49637 T	Man	Netherlands	DQ780343	EU570261	EU570269

Notes: *C.* = *Cladosporium*; Ex-type strains are indicated with the superscript "T", ex-epitype as "ET". The taxa obtained in this study are in bold. "–" indicates the absence of sequence data in GenBank.
